# Rapid Symptomatic Improvement and Long-Term Minimal Symptom Expression in a Myasthenia Gravis Patient Treated With Zilucoplan Without Maintenance Cholinesterase Inhibitors, Corticosteroids, or Immunosuppressants: A Case Report

**DOI:** 10.7759/cureus.102589

**Published:** 2026-01-29

**Authors:** Ryota Amano

**Affiliations:** 1 Neurology, Fujiyoshida Municipal Hospital, Fujiyoshida, JPN

**Keywords:** acetylcholine receptor antibody, c5 inhibition, minimal symptom expression, myasthenia gravis, zilucoplan

## Abstract

Myasthenia gravis (MG) is an autoimmune neuromuscular disorder, often requiring long-term immunosuppressive treatment. Although complement C5 inhibitors, such as zilucoplan, have shown robust efficacy in clinical trials, evidence of their use without maintenance cholinesterase inhibitors, corticosteroids, or other immunosuppressants remains limited.

In this report, I describe a woman in her late 60s with anti-acetylcholine receptor antibody-positive generalized MG, who achieved rapid symptomatic improvement and minimal symptom expression with zilucoplan. Clinical stability was sustained for at least one and a half years without concomitant cholinesterase inhibitors, corticosteroids, or other immunosuppressants. This case suggests that zilucoplan may represent an effective and durable treatment option for generalized MG.

## Introduction

Myasthenia gravis (MG) is an autoimmune neuromuscular junction disorder, most frequently associated with autoantibodies against the acetylcholine receptor (AChR), leading to fluctuating weakness of ocular, bulbar, limb, and respiratory muscles. Anti-AChR antibody-positive MG represents the most common subtype [1]. Although conventional therapies, such as cholinesterase inhibitors, corticosteroids, and other immunosuppressants, remain the mainstay of treatment, many patients experience incomplete disease control, treatment resistance, or substantial side effects. These limitations have prompted the development of targeted biologic therapies.

Currently, two major classes of mechanism-based drugs are available for generalized MG: neonatal Fc receptor (FcRn) inhibitors and complement C5 inhibitors. FcRn inhibitors, such as rozanolixizumab, efgartigimod alfa, and nipocalimab, accelerate the degradation of pathogenic IgG, thereby reducing circulating anti-AChR antibodies. Clinical trials have demonstrated their rapid onset of efficacy and favorable safety profiles in patients with generalized MG [2-4]. Complement C5 inhibitors, such as zilucoplan, eculizumab, and ravulizumab, represent another therapeutic strategy, as complement-mediated destruction of the postsynaptic membrane is a key driver of pathology. Zilucoplan, a once-daily subcutaneous macrocyclic peptide inhibitor of C5, has shown robust efficacy in the Phase 3 RAISE trial, achieving a placebo-corrected mean improvement in the Myasthenia Gravis Activities of Daily Living (MG-ADL) scale, with additional benefits in the quantitative MG (QMG) score [5-7]. Sustained benefits and long-term safety were further supported by the RAISE-XT extension study [8]. However, case reports describing the clinical use of zilucoplan remain limited, and, in particular, reports documenting rapid symptomatic improvement or disease control without concomitant cholinesterase inhibitors, corticosteroids, or other immunosuppressants are lacking.

In this report, I present a case of anti-AChR antibody-positive generalized MG in which rapid symptom improvement and achievement of minimal symptom expression (MSE), defined as pharmacologic remission (PR) or minimal manifestations (MM) according to the MGFA post-intervention status (MGFA-PIS), were obtained with zilucoplan, supported by an MG-ADL score of 0-1, without the need for maintenance cholinesterase inhibitors, corticosteroids, or other immunosuppressants.

## Case presentation

**Figure 1:**
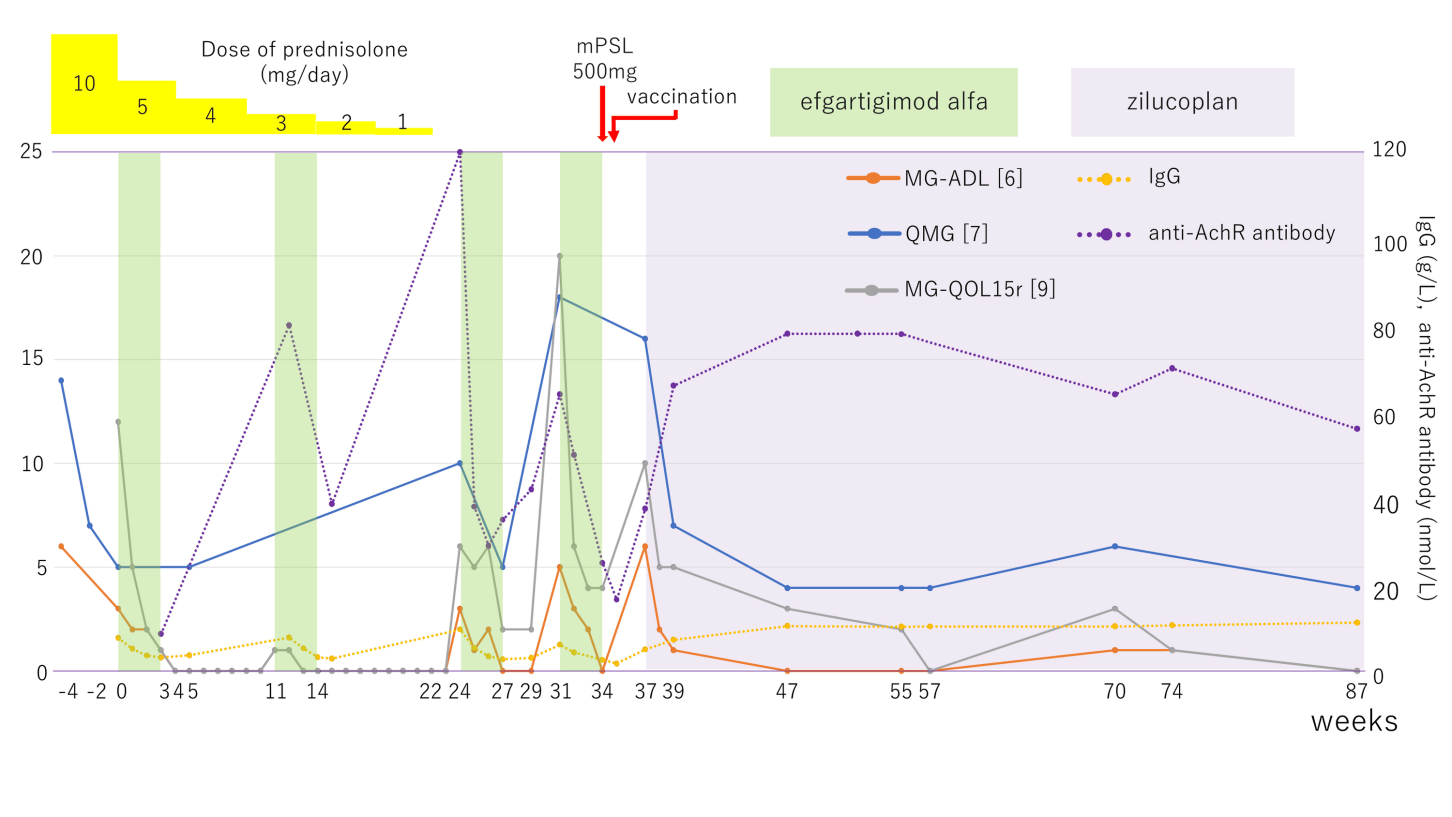
Timeline of the clinical course and changes in IgG and anti-AChR antibody titers after hospitalization for mPSL pulse therapy. Week 0 indicates the initiation of intravenous efgartigimod alfa, and zilucoplan was introduced at week 37. mPSL, Methylprednisolone; AChR, Acetylcholine Receptor; MG-ADL, Myasthenia Gravis Activities of Daily Living; QMG, Quantitative MG

A woman in her late 60s with no significant past medical history had been diagnosed with generalized MG (MGFA Class IIb) three years before the introduction of zilucoplan, initially presenting with ptosis, diplopia, and dysarthria. She was positive for anti-AChR antibodies, and no thymoma was detected on imaging; therefore, the diagnosis was late-onset MG. Her symptoms were controlled with oral prednisolone and pyridostigmine, without experiencing myasthenic crisis; however, she once discontinued prednisolone on her own because of concerns about adverse effects, which led to clinical worsening. For the first two years after disease onset, satisfactory symptom control was maintained with prednisolone and pyridostigmine. However, 41 weeks before the initiation of zilucoplan (-4 weeks in Figure 1), she was hospitalized because of worsening ptosis and diplopia. During hospitalization, high-dose intravenous methylprednisolone (mPSL) induced a transient exacerbation, followed by clinical improvement, and she was discharged after 12 days on prednisolone 10 mg/day (increased from 5 mg/day), with pyridostigmine discontinued.

Two weeks later, intravenous efgartigimod alfa was initiated for symptomatic control (week 0 in Figure 1). The response was excellent, and by one week after the first cycle, the MG-ADL and MG-QOL15r [9] scores were 0. She fulfilled the criteria for PR, according to the MGFA-PIS, thereby achieving MSE. A second cycle was administered at week 11, when mild ptosis reappeared. At the patient’s strong request, prednisolone was tapered to 5 mg/day the day after intravenous efgartigimod alfa initiation, and subsequently reduced by 1 mg every four or five weeks, reaching discontinuation by week 22.

From mid-week 23, she developed a rapid worsening of MG symptoms. Although conventional rescue options, such as restarting prednisolone, plasma exchange, or intravenous immunoglobulin, were considered, she strongly preferred antibody-directed therapy. Therefore, subcutaneous efgartigimod alfa with vorhyaluronidase alfa (efgartigimod SC) was initiated at week 24. This again resulted in rapid clinical improvement, with the MG-ADL score returning to 0 after one treatment cycle. However, symptoms recurred three weeks later, and an additional cycle was initiated at week 31. At week 34, she agreed to a single 500-mg infusion of intravenous mPSL to potentially prolong the therapeutic effect.

As described above, although efgartigimod alfa was highly effective and maintained an MG-ADL score of 0 while its therapeutic effect persisted, the limited durability of response with efgartigimod monotherapy - characterized by repeated cycles of short-term remission and relapse - represented the major treatment challenge. Accordingly, a treatment approach providing more sustained pharmacological activity, rather than intermittent, cycle-based administration, was deemed necessary.

In preparation for possible treatment escalation, and because she was unwilling to receive prednisolone or other immunosuppressive therapies, and because disease control with FcRn inhibitor monotherapy - including efgartigimod alfa - was considered insufficient, a treatment strategy involving the use of a C5 inhibitor at the time of the next exacerbation was planned. Accordingly, after providing thorough counseling regarding precautions for meningococcal infection, including the need for prompt medical evaluation and antibiotic treatment in the event of fever, she received a meningococcal (Groups A, C, Y, and W) conjugate vaccination at week 35.

**Figure 2:**
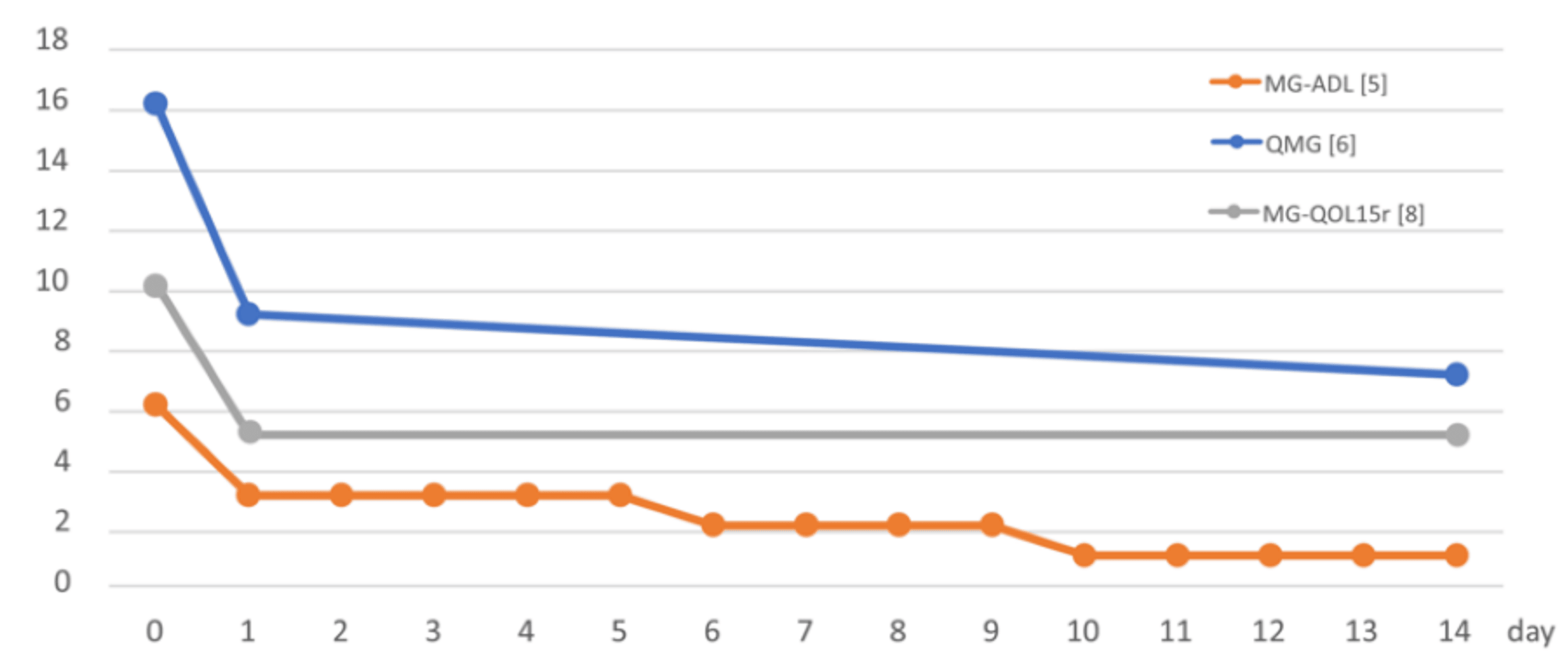
Clinical course during the first 15 days after initiation of zilucoplan. This panel illustrates detailed score changes from week 37 to week 39 in Figure 1. Zilucoplan was initiated on day 0, and clinical improvement was observed beginning on day 1. MG-ADL, Myasthenia Gravis Activities of Daily Living; QMG, Quantitative MG

Three weeks after the final dose of efgartigimod SC, her symptoms worsened, and she was classified as having an exacerbation (E) according to the MGFA-PIS. At that time, it was unclear whether disease control could be achieved with C5 inhibitors, and the possibility of combination therapy with efgartigimod SC was considered. Accordingly, among available C5 inhibitors, zilucoplan, a non-antibody agent, was selected. Zilucoplan (16.6 mg/day, administered subcutaneously) was initiated at week 37 without antibiotic prophylaxis. Baseline scores before treatment were QMG 16, MG-ADL 6, and MG-QOL15r 10 (day 0 in Figure 2). Remarkably, within five hours of the first injection, she reported symptomatic improvement, and by 24 hours, the scores had improved to 9, 3, and 5, respectively, suggesting a rapid onset of perceived benefit. By day 10, the MG-ADL score had improved to 1, and she met the criteria for MM according to the MGFA-PIS, corresponding to the achievement of MSE. Additionally, measurement of the classical 50% hemolytic complement (CH50) showed a level of 30 U/mL immediately before zilucoplan administration, which decreased to below the limit of detection (<10 U/mL) at 24 hours post-dose and remained below the limit of detection at day 14.

Thereafter, by week 47, her condition further improved, with scores of QMG 4, MG-ADL 0, and MG-QOL15r 3. She has since been followed for about one and a half years on zilucoplan, without maintenance cholinesterase inhibitors, corticosteroids, or other immunosuppressants. Between weeks 70 and 74 (Figure 1), she experienced mild, spontaneous worsening, without any identifiable triggers, but recovered without modification of treatment. At week 89, she returned to PR according to the MGFA-PIS, thereby maintaining MSE. No infections or injection-site reactions were observed to date.

## Discussion

In the present case, repeated cycle-based administration of efgartigimod, before the introduction of zilucoplan, consistently produced favorable clinical responses. When anti-AChR antibody titers were measured whenever feasible, the titers at the time of clinical worsening and initiation of efgartigimod alfa or zilucoplan at weeks 24, 31, and 37 were 120, 28.7, and 37.9 nmol/L, respectively. Although anti-AChR antibody titers appeared to increase around the time of symptom exacerbation, comparison using the QMG score, which is less influenced by patient subjectivity, showed scores of 10, 18, and 16 at weeks 24, 31, and 37, respectively. Notably, the highest antibody titer at week 24 was associated with the lowest QMG score, whereas the lowest antibody titer at week 31 corresponded to the highest QMG score. These findings suggest that, while anti-AChR antibody titers may appear to rise during periods of clinical worsening, a consistent correlation between antibody titers and clinical severity was not evident in this case.

After the initiation of zilucoplan, anti-AChR antibody titers did not show any abrupt increase and remained within a range of 56-76 nmol/L. Total serum IgG reached its lowest level at week 37 (4.99 g/L) following the initiation of zilucoplan, but recovered to 10.42 g/L by week 47, and subsequently remained stable, ranging from 10.27 to 11.23 g/L. Based on these findings, the direct antibody-reducing effect of the fourth cycle of efgartigimod alfa was considered to have diminished by at least week 47. 

Accordingly, although she experienced an unexplained, transient worsening after week 47, the overall maintenance of MSE thereafter is difficult to explain by a direct antibody-reducing effect of efgartigimod alfa. While a potential contribution from unknown, sustained clinical effects of intravenous mPSL or efgartigimod alfa cannot be excluded, the long-term maintenance of disease control in this case is most plausibly attributable to zilucoplan.

Anti-AChR antibodies are known to exert their pathogenic effects through three principal mechanisms: complement activation, receptor internalization, and blockade of the acetylcholine binding site. Previous studies have reported that, in a subset of patients, only complement activation is detectable as the dominant pathogenic mechanism [10]. In such patients, sustained clinical improvement with a C5 inhibitor, without concomitant cholinesterase inhibitors, corticosteroids, or other immunosuppressants, may be biologically plausible.

In recent years, several reports have described the use of FcRn inhibitors and complement C5 inhibitors as rescue therapies, particularly in patients with refractory or relatively severe MG. However, in all such cases, these agents were usually administered in combination with corticosteroids or other immunosuppressants. To date, published evidence remains scarce on clinical improvement with FcRn or C5 inhibitors, without concomitant cholinesterase inhibitors, corticosteroids, or other immunosuppressants. Two cases in which zilucoplan was used as rescue therapy have been reported [11,12]. In these cases, the extent to which the clinical improvement was attributable to zilucoplan alone versus synergistic effects with concomitant therapies could not be evaluated.

This patient demonstrated a rapid and marked clinical response to zilucoplan. Although her disease severity and background differed from those of previously reported critically ill patients, it cannot be ruled out that the fourth cycle of efgartigimod and the administration of intravenous mPSL (500 mg) at week 34 had some influence on the initial improvement between weeks 37 and 39; her clinical condition worsened between weeks 34 and 37. Moreover, no concomitant therapies were present at the time of zilucoplan initiation. Therefore, it is reasonable to consider that the primary factor responsible for the rapid clinical improvement observed within two weeks from week 37 was the pharmacological effect of zilucoplan. Furthermore, to my knowledge, there are no prior reports of long-term disease stability maintained exclusively with zilucoplan, without concomitant cholinesterase inhibitors, corticosteroids, or other immunosuppressants, for as long as one and a half years, as observed in this case.

In this patient, subjective symptom improvement began as early as five hours after the first administration of zilucoplan. Although a placebo effect cannot be completely excluded, according to the Pharmaceuticals and Medical Devices Agency (PMDA) regulatory review documents from the MG0009 Phase 2 study, pharmacodynamic studies have shown that zilucoplan achieves approximately 90% inhibition of complement-mediated erythrocyte hemolysis within three hours after administration [13], making the early onset of clinical improvement pharmacologically plausible. Furthermore, a significant improvement in the QMG score was observed on the day following treatment initiation. This assessment was performed by the same examiner, at the same time of day, and under identical conditions, and demonstrated clear, objective improvement.

Usman et al. reported a case series of three patients with myasthenic crisis requiring mechanical ventilation who were treated with eculizumab. Among them, one patient showed clinical improvement and was successfully extubated the day after eculizumab administration, while another achieved extubation within four days; in both cases, extubation was accomplished within one week [14]. Similarly, Ito et al. reported a case of myasthenic crisis in which successful extubation was achieved four days after the initiation of zilucoplan [11]. In addition, Yamanaka et al. reported the use of zilucoplan for preoperative disease control in a patient with thymoma-associated MG who had not progressed to myasthenic crisis. In that case, ptosis improved from the day following zilucoplan initiation, bulbar symptoms began to improve after three days, and within six days the QMG score decreased from 14 to 7 points and the MG-ADL score from 5 to 1 point [12]. Although not all patients with MG demonstrate such rapid or marked responses, these observations suggest that complement inhibition may exert relatively early therapeutic effects in selected patients. Further investigation is warranted to clarify which patient characteristics are associated with pronounced and rapid clinical improvement.

An observational analysis of the RAISE-XT trial population reported on changes in corticosteroid and immunosuppressant use following zilucoplan initiation [15]. Although this analysis included patients who were able to discontinue these agents after starting zilucoplan, detailed clinical courses were not available. Nevertheless, it is evident that a subset of patients - as in this case - can successfully discontinue corticosteroids while maintaining disease control, suggesting that such outcomes are not exceedingly rare.

## Conclusions

In conclusion, this case suggests that zilucoplan, administered without concomitant cholinesterase inhibitors, corticosteroids, or other immunosuppressants, may be effective in improving symptoms in MG and in sustaining clinical stability over a relatively long period.

However, this case has several important limitations. First, the observation was made in a non-blinded setting. Second, the evaluation is based on a single patient. Third, the patient had received efgartigimod SC and mPSL prior to the initiation of zilucoplan. Fourth, potential measurement error and learning effects associated with repeated QMG assessments cannot be excluded. Therefore, it is certainly not the case that all patients with anti-AChR antibody-positive MG can achieve adequate symptom control with zilucoplan, as observed in this case; indeed, some patients may exhibit minimal response or only mild improvement. Further studies are warranted to identify patient characteristics associated with durable disease control achieved with zilucoplan, administered without concomitant cholinesterase inhibitors, corticosteroids, or other immunosuppressants.
